# An evolutionary analysis identifies a conserved pentapeptide stretch containing the two essential lysine residues for rice L-*myo*-inositol 1-phosphate synthase catalytic activity

**DOI:** 10.1371/journal.pone.0185351

**Published:** 2017-09-26

**Authors:** Papri Basak, Susmita Maitra-Majee, Jayanta Kumar Das, Abhishek Mukherjee, Shubhra Ghosh Dastidar, Pabitra Pal Choudhury, Arun Lahiri Majumder

**Affiliations:** 1 Division of Plant Biology, Bose Institute (Centenary Campus), Kolkata, West Bengal, India; 2 Applied Statistics Unit, Indian Statistical Institute, Kolkata, West Bengal, India; 3 Bioinformatics Centre, Bose Institute (Centenary Campus), Kolkata, West Bengal, India; Weizmann Institute of Science, ISRAEL

## Abstract

A molecular evolutionary analysis of a well conserved protein helps to determine the essential amino acids in the core catalytic region. Based on the chemical properties of amino acid residues, phylogenetic analysis of a total of 172 homologous sequences of a highly conserved enzyme, L-*myo*-inositol 1-phosphate synthase or MIPS from evolutionarily diverse organisms was performed. This study revealed the presence of six phylogenetically conserved blocks, out of which four embrace the catalytic core of the functional protein. Further, specific amino acid modifications targeting the lysine residues, known to be important for MIPS catalysis, were performed at the catalytic site of a MIPS from monocotyledonous model plant, *Oryza sativa* (*Os*MIPS1). Following this study, *Os*MIPS mutants with deletion or replacement of lysine residues in the conserved blocks were made. Based on the enzyme kinetics performed on the deletion/replacement mutants, phylogenetic and structural comparison with the already established crystal structures from non-plant sources, an evolutionarily conserved peptide stretch was identified at the active pocket which contains the two most important lysine residues essential for catalytic activity.

## Introduction

The amino acids constituting a protein sequence are exposed to many evolutionary events over time. Some proteins are highly conserved across species while others have a high rate of evolution, indicating a low rate of homology among them. Intramolecular evolutionary studies help to determine relatedness of such orthologous genes belonging to distantly related species. The rate of evolution of a protein is calculated as an average of the rates obtained at an individual amino acid level where there is the presence of some evolutionary constrained regions indicating strong functionally important conservative zones [1].

L-*myo*-inositol 1-phosphate synthase (MIPS) or D-*myo*-inositol-3-phosphate synthase (EC5.5.1.4) is well known for its highly conserved core catalytic domain [2–4]. MIPS catalyses the first, rate-limiting redox reaction leading to the formation of free *myo*-inositol from D-glucose 6-phosphate [4]. *Myo*-inositol and its derivatives, phosphorylated or methylated function not only in the essential biological processes like membrane biogenesis, growth regulation, signal transduction etc. [5–7] but are also involved in various biotic and abiotic stress tolerance and defence mechanisms [8–10].

The evolutionarily conserved reaction pathway catalyzed by MIPS includes a series of oxidation, enolization, aldol cyclization and reduction step. In the presence of NAD^+^ as a cofactor at the catalytic site of MIPS, D-Glucose 6-phosphate is converted to *myo*-inositol 1-phosphate through two tightly bound intermediates, 5-keto-glucose-6-phosphate and *myo*-inosose 1- phosphate [11–13]. Studies on MIPS from *Archaeoglobus fulgidus* (*Af*MIPS) show that prokaryotic MIPS behave as a type II aldolase as they require divalent cations like Zn^2+^, Mg^2+^ or Mn^2+^ acting as a Lewis acid [14]. On the contrary, eukaryotic MIPS neither follow type-I aldolase reactions nor type-II, instead they need NH^4+^ ions. Basic amino acids at the catalytic domain of MIPS of *Saccharomyces cerevisiae*, particularly lysine residues, were assumed to be important for stabilisation of negative charges developed on carbon atoms of the substrate during and after enolization. Studies on yeast MIPS crystal structure bound with a high-affinity inhibitor mimicking the presence of substrate proved the importance of certain specific amino acids from the catalytic domain [15]. Substrate binding experiments were carried out by Neelon *et al*. [16] through site-directed mutagenesis of individual amino acid residues present at the catalytic site of *Af*MIPS which determined the importance of Lys274, Lys306, Lys367, Asn255 and Asp332 in interacting with Glucose 6-phosphate, *myo*-inositol 1-phosphate produced, NAD^+^ binding and NADH produced.

In this communication, an attempt is made to find out invariant conservation of certain amino acids amongst stretches of ‘conservative mutations’ regardless of their evolutionary diverse taxonomic positions. A total of six conserved blocks have been identified out of which four belong to the catalytic domain. Functional validation of the significance of two lysine residues within an evolutionary conserved pentapeptide stretch in the catalytic domain is achieved through *in vitro* mutagenesis of a MIPS from a monocot model plant, *Oryza sativa*, followed by the analysis of the change in functional activity of the mutants and further determination of its enzyme kinetics.

## Materials and methods

### Selection of MIPS orthologous sequences

In NCBI, a total of 2378 protein sequences are reported in bacteria, 257 in archea, 101 in protists, 233 in fungi, 534 in plants and 596 in animals. From these, all the partially encoded, hypothetical, predicted and unnamed sequences were eliminated. The MIPS sequences obtained were categorised according to biological classification proposed by Carl Woese *et al*. [17, 18].

For ease of study, those MIPS isoforms with more general length of protein sequences belonging to a particular kingdom were chosen. The sequences belonging to prokaryotes had more reports within the length of 355-380aa, 500-529aa for protists, fungi and plants, whereas 525-565aa for animals. There are many reports in NCBI indicating the same isoform but had different accession numbers, so in these cases only one was selected. In case of multiple reports from same species, isoforms belonging to particular strains with sequenced genome were chosen.

### Multiple sequence alignment and phylogenetic analysis of MIPS isoforms across evolution

To search for conserved amino acids, the protein sequences were subjected to MultAlin interface (http://multalin.toulouse.inra.fr/multalin/) using BLOSUM62. Alignment of the data was performed using ClustlW and phylogenetic analysis was carried out using MEGA 5.0 [19].

### Search for evolutionary conserved stretches

To search for the regions of conservative mutations across all the isoforms studied, the protein sequences were converted to numerical sequences as described in Das *et al*. [20], using numerical codes according to the biochemical nature of the side groups of individual amino acids. Group-1 consists of amino acids with acidic side chains- aspartate (D) and glutamate (E); Group-2 with basic- arginine (R), histidine (H) and lysine (K); Group-3 with aromatic- tyrosine (Y), phenylalanine (F) and tryptophan (W); Group-4 with aliphatic- alanine (A), glycine (G), isoleucine (I), leucine (L) and valine (V); Group-5 with cyclic- proline (P); Group-6 with sulphur-containing- cysteine (C) and methionine (M); Group-7 with hydroxyl-containing- serine (S) and threonine (T); and Group-8 with acidic amide side chains asparagine (N) and glutamine (Q).

The sequences were then subjected to MATLAB software to search for conserved stretches as tetramer, pentamer, hexamer, or longer conserved regions. Dissimilarity was calculated on the basis of occurrence of ‘non-conservative’ mutations with each of the individual conserved block, i.e. if group-4 (aliphatic) is replaced by group-3 (aromatic) at position 2 in a block containing 12 amino acid residues, then the dissimilarity percentage was calculated as (1/12)x100 = 8.33% and similarity% = 100 - (dissimilarity%).

### Amino acid modification studies of *Os*MIPS- a MIPS protein from a monocot model plant, *Oryza sativa*

Selective modification of the lysine residues of *Os*MIPS, transcribed from *OsINO1* gene isoform, Os03g0192700, was performed according to the method of Habeeb [21] with modifications as described. To find out the effect of lysine modification on MIPS activity, several parameters such as optimum trinitrobenzene sulphonic acid (TNBS) for complete modification, optimum time interval for complete modification and number of free amino groups available for trinitrophenylation were standardized initially.

The reaction mixture (1ml) containing 300 μl of 1.0 mg/ml purified protein in 20 mM Tris-Cl (pH 9.5), 590μl of 20 mM Tris-Cl (pH 9.5) and 110 μl of 0.1% TNBS (final concentration 0.011%) was incubated for 30 minutes at room temperature (25°C–30°C) in dark. 100 μl aliquots from the reaction mixture were withdrawn at different time intervals (5 mins upto 40 mins) and assayed for the residual MIPS activity after diluting it twice with MIPS assay buffer against proper control without TNBS.

To quantitate the number of free lysine residues in *Os*MIPS, 110 μl of 0.1% aqueous solution of TNBS was added to 300μl purified protein solution (1mg/ml, in 20 mM Tris-Cl, pH 9.5) in 590 μl of 20 mM Tris-Cl, pH 9.5 and incubated for 30 min in dark at 25°C. Then the reaction was terminated with 2.5% SDS and 0.1N HCl. A proper blank (minus enzyme set) as well as a negative control (without TNBS) were treated similarly. Thereafter, spectra of the samples were taken between 300 nm to 400 nm and absorbance plotted against the wavelength. As absorbance peaked at 367 nm, free amino group in the protein was calculated following the method of Habeeb [21] and Plapp *et al*. [22].

### Generation of site-directed *Os*MIPS mutants

Site-directed mutants of *Os*MIPS were constructed on the gene *OsINO1*, using Stratagene mutagenesis kit, as per manufacturer’s protocol [23]. The generated mutants and the specific primer sequences for each of them are shown in Table 1.

**Table 1:** Primers designed for *Os*MIPS mutations where each of the mentioned amino acid residues were either deleted or replaced by alanine.

**Deletion Mutants**			
			**Primer Sequence**
1	**ΔD342**	Forward	5'-ccacttggggaataatggcatgaacctttccgc-3'
		Reverse	5'-gcggaaaggttcatgccattattccccaagtgg-3'
2	**ΔL346**	Forward	5'-gatggcatgaactccgcacctcaaacattc-3'
		Reverse	5'-gaatgtttgaggtgcggagttcatgccatc-3'
3	**ΔK355**	Forward	5'-ctcaaacattccgatccgagatctccaagagc-3'
		Reverse	5'-gctcttggagatctcggatcggaatgtttgag-3'
4	**ΔK359**	Forward	5'-ccaaggagatctccagcaatgtggtcgatg-3'
		Reverse	5'-catcgaccacattgctggagatctccttgg-3'
5	**ΔV384**	Forward	5'-catcctgatcatgttgtgatcaagtatgtgccg-3'
		Reverse	5'-cggcacatacttgatcacaacatgatcaggatg-3'
6	**ΔI386**	Forward	5'-cctgatcatgttgttgtgaagtatgtgccgtatg-3'
		Reverse	5'-catacggcacatacttcacaacaacatgatcagg-3'
7	**ΔK396**	Forward	5'-gttggagacagcagggcaatggacgag-3'
		Reverse	5'-ctcgtccattgccctgctgtctccaac-3'
8	**ΔD422**	Forward	5'-caacacctgtgagtcactccttgccgcg-3'
		Reverse	5'-cgcggcaaggagtgactcacaggtgttg-3'
9	**ΔK468**	Forward	5'-cctgagctacctcaccgcaccccttgttc-3'
		Reverse	5'-gaacaaggggtgcggtgaggtagctcagg-3'
10	**ΔKEISK**	Forward	5’-caaacattccgatccagcaatgtggtcgatg-3’
		Reverse	5’-catcgaccacattgctggatcggaatgtttg-3’
**Substitution Mutants**			
			**Primer Sequence** *
1	**K355A**	Forward	5'-caaacattccgatcc*gcc*gagatctccaagagc-3'
		Reverse	5'-gctcttggagatctc*ggc*ggatcggaatgtttg-3'
2	**K359A**	Forward	5'-ccaaggagatctcc*gcc*agcaatgtggtcgatg-3'
		Reverse	5'-catcgaccacattgct*ggc*ggagatctccttgg-3'
3	**K396A**	Forward	5’-gttggagacagc*gcc*agggcaatggacgag-3’
		Reverse	5’-ctcgtccattgccct*ggc*gctgtctccaac-3’
4	**K468A**	Forward	5’-ctgagctacctcacc*gcc*gcaccccttgtt-3’
		Reverse	5’-gaacaaggggtgc*ggc*ggtgaggtagctcag-3’

*Italicised and underlined nucleotides encoding alanine were used for site-directed mutagenesis to substitute lysine for alanine.

### Expression and purification of wild *Os*MIPS and the *Os*MIPS deletion/substitution mutants

All the deletion mutants except ΔKEISK, were raised on *OsINO1*/pT7-7 clone, a bacterial expression vector. Mutants were transformed in *E*. *coli* BL21 (DE3) strain. The wild type and the mutant proteins were overexpressed following an overnight induction with 0.5 mM IPTG at 20°C. The cells were harvested and sonicated (4 x 20 sec pulse at 250 W) in homogenization buffer (20 mM Tris-Cl pH 7.5, 10 mM NH_4_Cl, 20% glycerol, 8 mM β-ME and 2 mM PMSF). The homogenate were centrifuged at 15000 rpm for 30 mins at 4°C. The wild type and the mutant proteins were expressed mostly in the soluble phase at variable ratio. The expressed proteins were purified to near homogeneity through DEAE Sephacel ion exchange column chromatography [3]. Protein was allowed to get adsorbed to the matrix, pre-equilibrated with buffer A containing 20 mM Tris-Cl pH 7.5, 10 mM NH_4_Cl, 2 mM PMSF, 8 mM β-ME and 20% glycerol. Bound proteins were eluted with a continuous linear gradient of NH_4_Cl between 100 mM to 250 mM of running buffer A. All fractions (1ml each) were assayed for MIPS activity. The fractions showing maximal enzyme activity were pooled and dialyzed against 500ml of buffer A.

For expression and purification of *Os*MIPS substitution mutants the lysine residues present within the core catalytic domain at positions K355, K359, K396 and K468 of *Os*MIPS were substituted by alanine using primers containing mutated sites (Table 1). Double mutant (K355A, K359A) was prepared using K355A and/or K359A single mutants using primers K359A and K355A respectively. In addition, a pentapeptide deletion mutant (ΔKEISK) was also raised. NEB’s Q5 High-fidelity DNA polymerase was used for whole plasmid amplification of *OsINO1* cDNA cloned to pET15b using the designed primers, followed by Dpn1 digestion to digest the methylated parental strands and transformed into BL21 (DE3) expression host. *Os*MIPS as well as the mutant proteins were induced using IPTG and were expressed in the pellet fraction. The proteins solubilised from the pellet fractions were purified through Ni-NTA column equilibrated with column equilibration buffer (50 mM NaH_2_PO_4_, 300 mM NaCl and 10 mM imidazole; pH 8.0). Bound proteins were eluted using a continuous linear gradient of imidazole between 50 mM and 250 mM. The eluted protein fractions were checked on 10% SDS-PAGE for purity and the fractions containing higher concentration of purified proteins, from 50 mM, 100 mM and 150 mM fractions were pooled and dialysed to remove the imidazole content completely.

### Functional activity and enzyme kinetics of the mutant *Os*MIPS enzymes

The mutant enzymes were assayed colorimetrically by periodate oxidation method of Barnett *et al*. [24]. The assay mixture contained 50 mM Tris-HCl, pH 7.5, 14 mM NH_4_Cl, 0.8 mM NAD^+^, 5 mM β-ME, 5 mM glucose-6-phosphate and enzyme in a total volume of 0.5 ml. Suitable blanks without an enzyme were taken as control. After incubation at 37°C for 1 hour, the reaction was terminated by heat denaturation. The reaction product, inositol-1-phosphate (I-1-P) was oxidized using sodium periodate at 37°C for 1 hour and then quenched with Na_2_SO_3_. Released inorganic phosphate was determined colorimetrically by the method of Chen *et al*. [25]. The MIPS activity determination was further corroborated by L-*myo*-inositol 1-phosphate phosphatase (I-1-Pase) assay [3] using either purified rat testes I-1-Pase or purified bacterially expressed, *sll1383* gene product from *Synechocystis* sp. PCC 6803 [26]. Km and Vmax values of the enzymes for NAD^+^ and G6P were calculated following standard procedures.

### Structural analysis of conserved amino acid residues

To find out the pattern of positioning of individual conserved amino acid residues in the catalytic domain and their interactions with the substrate or cofactor were labelled onto the yeast crystal structure, 1RM0 using PyMOL (https://www.pymol.org/).

Each mutant was solvated using a cubic water box ensuring at least 9Å thickness of water layer everywhere. CHARMM36 force field [27] was used to represent the system. After energy minimization, each system was heated to 300K and equilibrated; then it was followed by molecular dynamics simulation run for 10ns at constant temperature and pressure using NMAD package [28]. The integration time-step was set to 2fs and SHAKE was applied to freeze the vibration of the bonds involving hydrogen. PME [29] was used to compute the long ranged interactions whereas the short ranged interactions were truncated at 12Å.

## Results and discussion

### Selection of MIPS homologous sequences across all forms of life

According to the three-domain system of biological classification proposed by Carl Woese *et al*. [17], there are 3 domains of life on the basis of differences in 16S rRNA—Archaea, Bacteria and Eukaryota. The domain eukaryote, thought to be evolved from archea, is further divided into 4 kingdoms- Chromalveolate or Protista, Plantae, Animalia and Fungi. Organisms belonging to the domains archaea and bacteria are prokaryotic in nature. Although archea and bacteria had diverged from a common ancestor, the thermophilic bacterial species belonging to the phylum Thermotogae, are reported to have derived their *INO1* genes through horizontal gene transfer from archea [17, 18, 30, 31].

Out of numerous reports for MIPS homologous sequences, 6 in bacteria, 38 in archea, 69 in protists, 28 in fungi, 40 in plants and 36 in animals were finally selected for further evolutionary analysis. All the 172 MIPS homologous sequences selected are enlisted in S1 Table. For the sake of simplicity, the bacterial sequences have been represented as Ba1-Ba6, archeal sequences as Ar1-Ar38, protist sequences as Pr1-Pr24, plants as Pl1-Pl40, animal sequences as An1-An36 and fungal sequences as Fu1-Fu28. The sequences from protists, fungi and plants had their lengths mostly within the range of 500–529 aa and so only the MIPS protein sequences falling within this range were chosen. A wider range of protein length 525–565 aa was chosen for the case of kingdom Animalia to include the MIPS sequences of some remarkable model organisms like *Caenorhabditis elegans* (525 aa), *Xenopus tropicalis* (564 aa) and *Drosophila melanogaster* (565 aa). On the contrary, prokaryotic MIPS had a much smaller length of the translated product than eukaryotes, mainly ranging from 360–369 aa. From archaea, a total of 38 sequences of only those organisms with their complete genome sequenced and within the range of 355–380 aa were selected. Out of a large number of reports for bacterial MIPS, the isoforms from only 6 representative organisms were selected *viz*. *Clostridium* sp. D5, *Streptomyces griseus* subsp. *griseus*, *Bacillus thuringiensis* subsp. *indiana*, *Mycobacterium tuberculosis*, *Rhizobium leguminosarum* and *Thermotoga maritima*.

### Large scale phylogenetic analysis of the MIPS sequences across evolution

**Fig 1:**
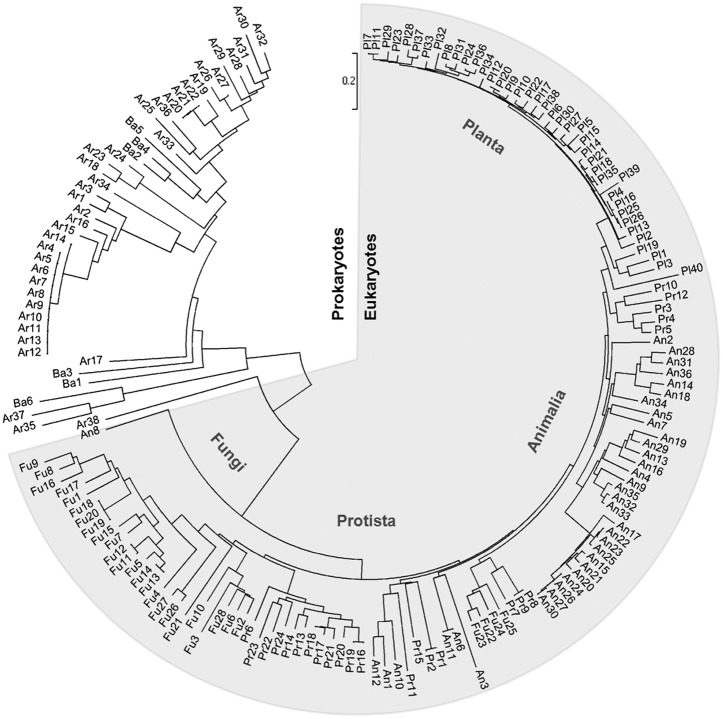
Evolutionary relationships of taxa for all 172 MIPS sequences. Prokaryotes and Eukaryotes have a well-defined boundary. Eukaryotic sequences have been denoted by shaded zone. The evolutionary history was inferred using the Neighbor-Joining method [32]. The optimal tree with the sum of branch length = 18.55289526 is shown. The tree is drawn to scale, with branch lengths in the same units as those of the evolutionary distances used to infer the phylogenetic tree. The evolutionary distances were computed using the Poisson correction method [33] and are in the units of the number of amino acid substitutions per site. The analysis involved 172 amino acid sequences. All positions containing gaps and missing data were eliminated. There were a total of 222 positions in the final dataset. Evolutionary analyses were conducted in MEGA5 [18].

Phylogenetic analysis was performed using neighbour-joining method (MEGA 5.0) for the 172 sequences under study. As shown in Fig 1, the prokaryotic homologs are phylogenetically well separated from eukaryotic MIPS with an average evolutionary distance of 0.918. The MIPS homologs belonging to kingdom planta show the most conserved profile while animal homologs are found to group with 11 out of 24 of the protist homologs and few of fungal homologs (Fu22-25). An8, MIPS from *Fundulus heteroclitus*, a bottom-dwelling fish species known to survive well in high range of salinity, temperature, water hardiness and oxygen fluctuations, is however much more diverse and is found to form the outgroup amongst eukaryotes. Fungal sequences too form a separate domain except the four grouping with animal homologs as mentioned above. They include the two isoforms from *Mortierella verticillata* and single MIPS from *Rhizophagus irregularis* and *Conidiobolus coronatus*.

Based on crystal structure of *Saccharomyces cerevisiae* MIPS, Stein and Geiger [34] reported that MIPS has three main domains- central domain, NAD-binding or Rossmann fold domain (residues 66–326) and the catalytic or tetramerization domain (residues 327–441). Prokaryotic MIPS are shorter by 60–80 aa than eukaryotes at the N-terminus. Although the active sites of NADH-bound *Sc*MIPS and NAD^+^-bound mycobacterial MIPS crystal structures are similar, *Mycobacterium tuberculosis* MIPS lacks N as well as C terminal domains corresponding to the central domain; thus the sequence begins with NAD^+^-binding domain but lacks the three important Rossman-fold insertions [35, 36].

### Sequence comparison at a large scale on the basis of chemical properties of individual amino acids

There are a total of 23 proteinogenic amino acids out of which 20 are the ‘standard’ ones encoded by triplet codons of the mRNA. All the α-amino acids have different side chains or R groups attached to the α-carbon atom and so they can be classified on the basis of their side chains. Here this principle has been followed for a large scale evolutionary analysis to figure out the significant invariably conserved amino acids with flanking stretches of permissible conservative mutations irrespective of its taxonomic position. As mentioned in the Materials and Methods section, all the protein sequences were converted numerically using MATLAB according to the eight classes of chemical nature of the side chains of individual amino acids and alignment was carried out accordingly as described by Das *et al*. [20].

**Fig 2:**
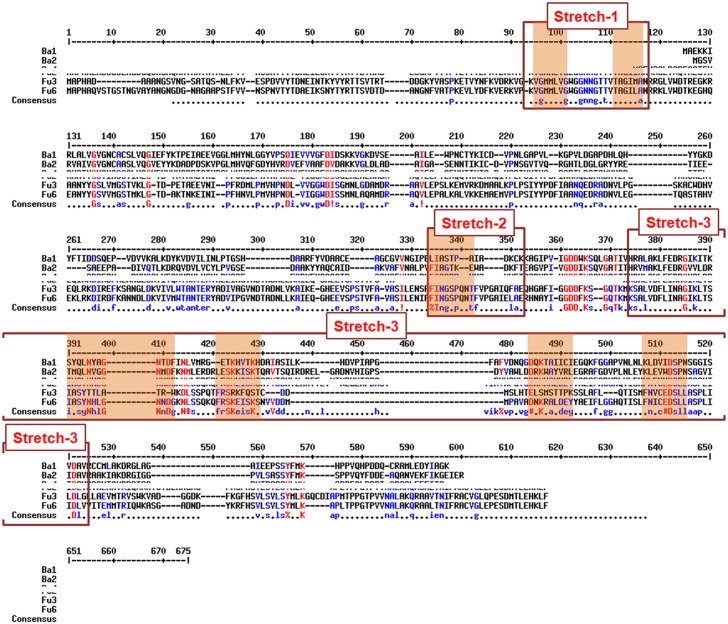
Conserved region identified in MultAlin of all the 172 MIPS sequences selected. Long stretches found to be phylogenetically conserved are shown in the figure. The red shaded zones within the denoted stretches represent the six phylogenetically conserved blocks present in eukaryotes. Stretch-1 consists of only block-F with two different sites of occurrence.

Initial search for conserved patterns throughout 172 selected sequences across evolution compromising the similarity parameters to as low as 30% to as high as 100% resulted in determination of three large conserved stretches. Results presented in Table 2 reveal that Stretch-1(32 amino acids) and Stretch-2 (23 amino acids), both belong to Rossmann-fold domain whereas the largest one, Stretch-3 consisting of 128 amino acids span the entire catalytic domain. Prokaryotes maintain a similarity of 43–57% with Stretch-2 and less than 38% with stretch-3. Range of occurrence of the three large conserved stretches have been depicted as MultAlin of all the 172 sequences studied (Fig 2). Loci studies of Stretch-1 show that the most of the eukaryotic sequences having this stretch start from 55-75aa corresponding to the initial amino acids within NAD^+^ -binding domain while prokaryotes show similar stretches in multiples at positions which are not conserved among themselves and are found in the middle of the NAD^+^ -binding domain. It is to be noted that, this study was performed on converted data and so such similar sequences also appeared elsewhere along the whole length of protein sequences.

**Table 2:** Conserved large stretches and blocks found among the MIPS homologous sequences.

Conserved Stretches	Domain	Converted sequences ^a^	Blocks Involved
Stretch-1	NAD^+^—binding	45244464443448847747444448224473	Block-F (F.1 and F.2)
Stretch-2	NAD^+^—binding	34847588734546414444444	Block-E
Stretch-3	Catalytic	274441344444425774473824488144847	Block-D
		458732721472784411644787443454125	Block-C
		126444234534417224611377143644287	Block-B
		44287611744445444144444144724	Block-A

The three large conserved stretches found across 172 homologous sequences with similarity ≥30%, represent the two important domains of MIPS quaternary structure. The conserved blocks within eukaryotic sequences with similarity ≥70%, named as blocks A, B, C, D E and F are underlined.

^**a**^ The numbers from 1 to 8 in the converted sequences refer to the amino acids falling under Groups 1 to 8 as described in Materials and Methods, and Das *et al*. [20].

**Fig 3:**
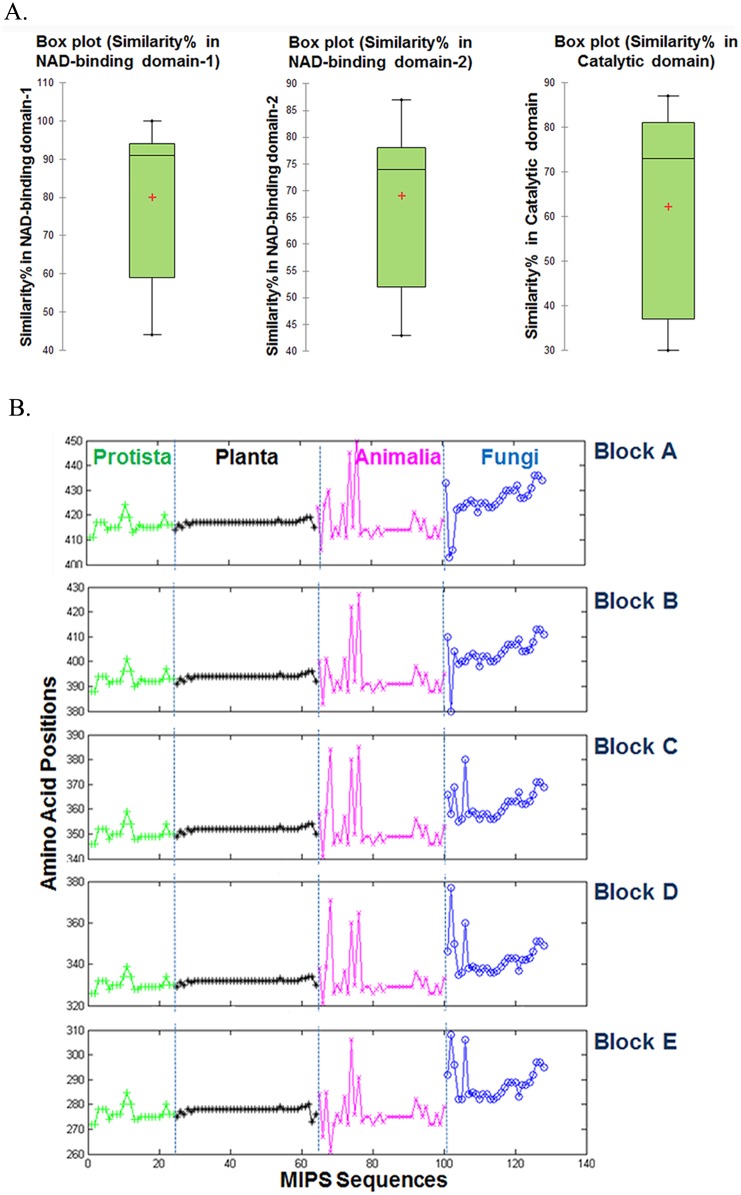
Homology stretches based on similarity percentage. (A) Box- plot for Similarity % of three large conserved stretches across evolution. The horizontal line within each box denotes median values, ‘+’ sign denotes the mean value. (B) Loci of Blocks A to E are compared among the selected eukaryotic MIPS sequences.

While considering the three large stretches identified, box-plots in Fig 3A depict that most of the sequences occur above median and thus show high homology for individual stretches. Another scanning for longest possible conserved stretches was performed but this time conserved blocks with similarity more than 70% were considered and thus the prokaryotic sequences got eliminated in this procedure. Among all the 128 eukaryotic sequences studied, fungal sequences were evolutionarily much diverse than the sequences from Protista, Planta or Animalia which maintained an overall high sequence similarity. A total of six blocks (Table 2) were found to be most conserved among all the eukaryotic kingdoms studied and have the length of 7 to 12 amino acids: Block A- 28761144, Block B- 172246113, Block C- 327214727, Block D- 447382448814, Block E- 348475887 and Block F- 4444444. The loci of their occurrence along the protein length were found to be present within the large conserved stretches mentioned earlier and they have been depicted in as red bordered boxes in Fig 2B. Block-F has multiple sites of occurrence in many sequences but mostly twice within the stretch-1 having a spacer of 8 amino acids. For the rest of the conserved blocks, sites of occurrence along the full length protein sequence were generated from MATLAB and are shown in Fig 3B. Blocks with only single site of occurrence i.e. Block A to E (Table 2) have been selected.

### Invariant amino acids found within the six highly conserved patterns

Detailed analysis of the identified conserved blocks has been performed for the 128 eukaryotic MIPS protein sequences through MultAlin analysis and was compared with the 44 prokaryotic sequences.

#### Block A

Majority of the sequences belonging to Block A-287611744 are HNTCEDSLL. A few from protists and animals viz. Pr(6, 22–24); An(3,4); including fungal sequences Fu(1, 5, 7, 11–15, 17–20, 22–25) share the sequence with 11% dissimilarity. Pr(6, 22–24) have the sequence as HNTCQDSLL, An3 has YNTCEDSLL, An4 HNMCEDSLL, Fu(1, 5, 6, 11–15, 17) have HNVCEDSLL, Fu(18, 19) have HNICEDSLL, Fu(22–25) have it as YNTCEDSLL. Loci are generally within 410–440 aa. Prokaryotes have only 50% similarity with this block. From MultAlin, the block-A is present as h-N-t-C-E-D-S-L-L among eukaryotes while it is x-n-x-c-x-D-s-l-l considering both the prokaryotic and eukaryotic sequences, where amino acids denoted in capital letters indicate >90% consensus, small letters indicate 50–90% consensus and ‘x’ represent any amino acid. Thus, throughout evolution, the aspartic acid (Asp) at position no. 6 i.e. #6 of Block-A is invariably maintained while conservative mutations have been allowed in the flanking sites. This indicates a strong correlation of the presence of ‘the’ Asp and the conservation of catalysis of MIPS.

#### Block B

The amino acid stretch, DSKRAMDEY in particular, i.e.172246113 is 100% conserved in plants, 22/36 and animals, 17/28 in protists. Rest of the 13 animals and 9 protists have a leucine instead of methionine at position no. 6 (i.e. #6) and thus have the sequence DSKRALDEY representing 172244113. Though the #6 is being occupied by amino acids from different groups, the sequences from all protists, plants and animals with respect to Block-B are 90% conserved as 9 out of 10 sites are identical. This block is conserved 100% with M at #6 in 16/28 fungal sequences, 5/28 Fu(21–25) are with DSKRALDEY, 9/28 have DSKVAMDEY and 2/28 have DSKIAMDEY. Fu3 referring to *Candida tropicalis MYA-3404* is an outgroup here having the block as EGKRAANYY in a maximum-likelihood (ML) tree (data not shown) with only 36.36% similarity. MultAlin results reveal that D-s-K-r-A-m-D-E-Y is conserved among eukaryotes while x-x-K-x-a-x-d-e-y is conserved throughout all the 172 selected sequences. Thus, within the conserved stretch of Block-B, we find yet another invariant amino acid, lysine (Lys) at #3, flanked by a total of 8 amino acid positions that allow conservative mutations.

#### Block C

This block (327214727) is mostly conserved as FRSKEISKS forming the main phylogenetic domain. Fu3 (*Candida tropicalis* MYA-3404) with 22% dissimilarity again forms the outgroup in the ML-tree. Disparity is shown at #6 with the presence of valine instead of isoleucine for animal sequences- An(15, 17, 20, 21, 24, 26, 27 and 30). 8 out of 28 protist sequences have FRSKEITKS as Block-C. Collectively, this block is highly conserved in eukaryotes with occurrence of F-R-S-K-E-x-s-K-s while both the prokaryotes and eukaryotes show conservation of this block as f-r-S-K-e-i-s-K-x. Thus, a serine at #3 and two lysine residues at #4 and #8 are the three invariant amino acids comprising the conserved Block-C.

#### Block D

Biggest conservative block found is a 12-mer- 447382448814: Among eukaryotes, the 12-mer block in most of the plants, animals and 11 out of 24 protists, Pr(1–5, 7–12), have the stretch as IVSYNHLGNND. It is to be noted the 12-mer stretch from the 11 protists, is the same as the higher, much evolved multicellular organisms. Exceptions among plants are Pl2 belonging to *Oryza sativa* (Accession No.-BAT10553.1) and Pl40 from *Bathycoccus prasinos* (Accession No. XP_007509183.1); *Drosophila melanogaster* MIPS from animals; and protist sequences Pr(6, 13–24). They have alanine instead of valine at #2, IASYNHLGNNDG, although both Alanine and Valine fall within Group 4 i.e. aliphatic. 22 out of 28 fungal sequences, Fu(1, 2, 4–20, 26–28), shared the same block while rest of them have ITSYNHLGNNDG. Here, at #2, group 4 is completely replaced by hydroxyl- containing threonine. However, Fu21 (from *Mortierella verticillata* NRRL 6337) with IASYNHLGMPFF and Fu3 (MIPS from *Lodderomyces elongisporus*) with VLSYMLKGQCDI form the outgroups. The Block-D is the first stretch of conserved block in the catalytic site of MIPS protein sequence (Fig 2B) and this is represented in eukaryotes as I-v-S-Y-N-H-L-G-N-N-D-G, completely conserved at 11 out of 12 amino acid positions. While considering prokaryotes as well, we still find a high conservation of Block-D as i-x-s-y-N-h-l-G-N-n-D-g. Thus, here we find 4 invariant amino acids, two asparagines (Asn), a glycine (Gly) and an aspartic acid (Asp).

#### Block E

Loci studies with Block-E denote the block to be present within 275–285 aa stretch i.e. NAD-binding domain of MIPS tertiary structure. In this case, all the sequences of 348475887 in protists and plants (except Pl39) are 100% identical with block E. Most common sequence is F/YI/VNGSPQNT for protists and animals whereas plants have the block as FINGSPQNT invariably. Pl39 (MIPS from *Porteresia coarctata)* was found to lack the Block-E at the designated position ~270aa. Among the fungal MIPS, Fu2 this block is missing and Fu4 has the sequence as FINGSPSIL with 33% dissimilarity with respect to the amino acid grouping we followed. The block-E is conserved across eukaryotes as F/Y-I/V-N-G-s-P-Q-N-T while across evolution it is conserved as F/Y-I-n-g-x-p-x-x-t. Thus, an aromatic amino acid always occurs at #1 (phenylalanine/tyrosine) and isoleucine is invariant at #2 of Block-E.

#### Block F

Occurrence of 4444444 in eukaryotes is mostly at two positions as l-G-v-L/M-L/M-I/V-G and l-t-a-g-x-x-A separated by 8 amino acids. The last glycine residue of the Block-F.1 is the first G of the signature motif of Rossmann fold, GwGGNNG, present only in eukaryotes. Protists mostly have the F.2 in their sequences while fungal sequences have representation of 7 consecutive aliphatic amino acids in multiples sometimes, overlapping. Prokaryotes, however, do not have the Block-F.1, F.2 or GwGGNNG motif. In their case, 4444444 is present within the first 10 or 15 amino acids v-a-i-x-G-x-G. Thus, invariant amino acid throughout evolution for Block-F is absent.

### Comparing the six conserved patterns established with reported crystal structures

The importance of the protein sequence patterns on the structure of specific regions can be detected by aligning them to a protein of known structure. Till date, crystal structure of MIPS from only 5 organisms are submitted in RCSB PDB—*Saccharomyces cerevisiae* (Fungi), *Archaeglobus fulgidus* (Archea), *Mycobacterium tuberculosis* (Bacteria), *Thermotoga maritima* (Bacteria) and *Caenorabditis elegans* (Animalia). Many crystal structures with cofactor, substrate and inhibitor bound crystals have been proposed. No crystal structure has been reported from kingdom planta yet. MIPS from a plant model, *Oryza sativa* (BAS82735.1) was chosen for justifying the functional significance of evolutionary conserved amino acid blocks. The analysis performed revealed a few highly conserved ‘invariant’ amino acids throughout evolution and based on homology searches, these residue positions were compared with that of established crystal structures (Table 3).

**Table 3:** Invariant amino acids found within the highly conserved patterns in eukaryotes and comparing them with the established crystal structures.

Conserved blocks	Amino acid sequence ^a^	*Mt*MIPS ^a^	*Tm*MIPS ^a^	*Af*MIPS ^a^	*Sc*MIPS ^a^	*Ce*MIPS ^a^	*Os*MIPS ^a^
Block A	xnxcxDsll	klevwD_310_sPN	grinD_316_spa	iwdaiD_332_aiv	hnvceD_438_sll	hntceD_428_sll	hntceD_422_sll
Block B	xxKxaxdey	drK_284_wayvr	dkK_290_fiaihi	dnK_306_tafdfv	dsK_412_vamdey	dsK_402_ramdey	dsK_396_ramdey
Block C	frSKeisKx	leS_247_K_248_kisK_252_t	nkS_256_K_257_eftK_261_s	keS_273_K_274_vlsK_278_d	frS_368_K_369_eisK_373_s	frS_360_K_361_eisK_365_s	frS_354_K_355_eisK_359_s
Block D	ixsyNhlGNnDg	tmqlN_229_vgG_232_N_233_mD_235_f	vaqfN_238_igG_241_N_242_mD_244_f	wmsyN_255_ilG_258_dyD_261_g	iasyN_350_hlG_353_N_354_nD3_56_g	ivsyN_342_hlG_345_N_346_nD_348_g	ivsyN_336_hlG_339_N_340_nD_342_g
Block E	F/YIngxpxxt	F_168_vnalpvfi	FI_187_andpa	Y_196_anftpspgs	YI_293_ngspqnt	YI_285_ngspqnt	FI_279_ngspqnt
Block F	F.1: lGvL/ML/MI/VG and F.2: ltagxxA	-	-	-	lG_67_iMLIG_72_ tvasvlA_87_	tGlLLVG_71_ avgsifA_86_	lG_63_vMLVG_68_ ltagviA_83_

*Mt*- *Mycobacterium tuberculosis*, *Tm- Thermotoga maritima*, *Af- Archaeglobus fulgidus*, *Sc- Saccharomyces cerevisiae and Ce- Caenorabditis elegans*. *Os*MIPS is the plant model for comparison as there is no plant MIPS crystal structure reported till now.

^**a**^ The underlined amino acids represented in upper case have ≥90% similarity across all 172 sequences studied. Locus of the individual residue in the protein sequence is denoted as subscript. The residues represented in upper case only have 50% similarity for that position. Lower case represents ≤50% similarity.

Detailed studies on yeast crystal structures followed by site-directed mutagenesis studies on archeal MIPS catalytic sites revealed involvement of ‘key amino acids’ at the catalytic site for MIPS catalytic activity [16, 36, 37]. The aspartic acid of Block-A was found to be involved in interaction with a second divalent cation, for proper orientation of NAD^+^ along with lysine of Block-B. The first lysine of Block-C is involved in stabilisation of the O5 during enolization while the second lysine is important for NAD^+^ binding and its reduction. The first asparagine of Block-D was found to be involved in strengthening G6P binding to the catalytic site.

### Importance of lysine residues at the catalytic domain of MIPS

During the entire catalysis, a close proximity of four lysine residues in *Af*MIPS present at the active site, is thought to be involved in protonotion at various centres like, O1 and O5 and act as a base during deprotonation [37]. Keeping this in mind, several basic amino acid residues such as histidine (H), arginine (R) and lysine (K) were modified by established procedures [21, 38, 39]. Such experiments revealed that selective modification of either H or R does not alter the activity of MIPS, suggesting a possible non-involvement of these residues in the actual MIPS catalysis (data not presented). However, a steady decrease in the MIPS activity was observed with time as Lys modification progressed, which indicates a probable involvement of K residues in the MIPS catalysis. The number of free amino group (K) available for modification, which in turn will give the probable number of K residues that might be involved in the actual catalysis, was determined (S1A Fig). In this case, 6 lysine residues out of the 31 in *Os*MIPS had been found to be accessible for modification. When purified MIPS was subjected to selective lysine modification by TNBS, a fast inactivation of the enzyme was noticed, the enzyme activity dropping to ~90% in about 40 seconds (S1B Fig).

### MIPS functional activity of *Os*MIPS Lys mutants

Following the observation that Lys modification of MIPS protein adversely affects the enzyme activity, amino acid modification studies involving the evolutionary conserved Lys residues of the MIPS sequences were attempted as observed in S1 Fig and Table 3. Prior to such experimentation, to have a quick assessment of the possibility of deletion-induced structural changes of the molecules, two of the ‘deleted’-variants of the protein (*viz*. ΔK355 and ΔK359) were computationally modelled in all atom description and each was allowed to relax their internal strains during standard molecular dynamics simulation run for 10ns. Some local fluctuations and microscopic re-adjustment of interactions were noticed but the overall protein structure in their tetrameric form was stable in both cases. Therefore, it could be inferred that the deletion of the residues did not generate any major internal strain to distort the tetramer and the minor perturbations were absorbed locally; so these ‘deleted’-variants serve as the valid systems for the experimental investigations. To validate this, further experimental investigations were performed with a few mutations having replacement of lysine by alanine, to compare them with the deletions.

**Fig 4:**
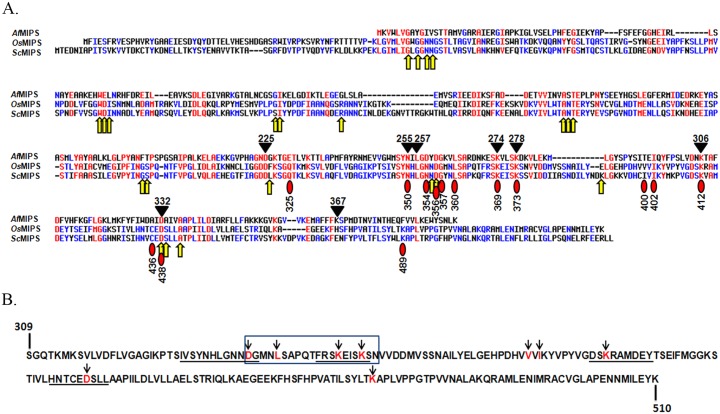
Construction of *Os*MIPS deletion/substitution mutants. (A) Sequence alignment comparison study of *Os*MIPS, *Sc*MIPS and *Af*MIPS through MultAlin. Yellow arrow signifies the NAD-binding residues; red oval signifies the substrate binding residues in *Sc*MIPS; and black triangle active site residues in *Af*MIPS. Numbering of amino acids presented at the bottom line is with respect to *Sc*MIPS sequence and numbering of amino acids presented at the top line is with respect to *Af*MIPS sequence. (B) Diagrammatic representation of the generated site-specific deletion mutants of *Os*MIPS. Black arrows point to the amino acid residue (in red font) has been deleted and/or replaced by alanine from the wild-type *Os*MIPS following the procedure described in Materials and Methods section. The box represents the “core catalytic domain” and the four evolutionarily conserved blocks within the catalytic site are underlined.

On the basis of crystal structure information of *Sc*MIPS and *Af*MIPS on the probable active site amino acids, selected *in vitro* mutagenesis of the important amino acid residues including lysine was attempted to evaluate the role of these in *Os*MIPS catalysis (Fig 4A). Mutants of *Os*MIPS were generated on bacterial expression vectors at selected sites in the catalytic domain as indicated in Fig 4B. The evolutionary conserved Lys residues (K355, K359, K396 and K468) were either deleted or replaced by Ala. The wild type *Os*MIPS and all the mutants were raised in pT7 or pET15b vectors including the deletion mutant ΔD342-N361, which lacks the catalytic core region [2, 3]; the pentapeptide deletion mutant ΔKEISK, targeting the proposed pentapeptide; and the double mutant K355A,K359A, targeting both of the two significant lysine residues within the pentapeptide. Bacterially expressed MIPS proteins from *Oryza* (termed *Os*MIPS), purified upto homogeneity through procedures published earlier from this laboratory [3] or as described in Materials and Methods were used for the amino acid modification experiments. Most of the deletion mutants were expressed in soluble phase in variable degrees except ΔK468 and all the substitution mutants, a considerable portion of which was expressed in the particulate fraction as judged by SDS-PAGE analysis (data not shown). The MIPS proteins expressed in pET15b vectors were purified through procedures described in Materials and Methods. However, all of them showed immunoreactivity towards the antibody against purified *Os*MIPS protein (data not shown).

**Fig 5:**
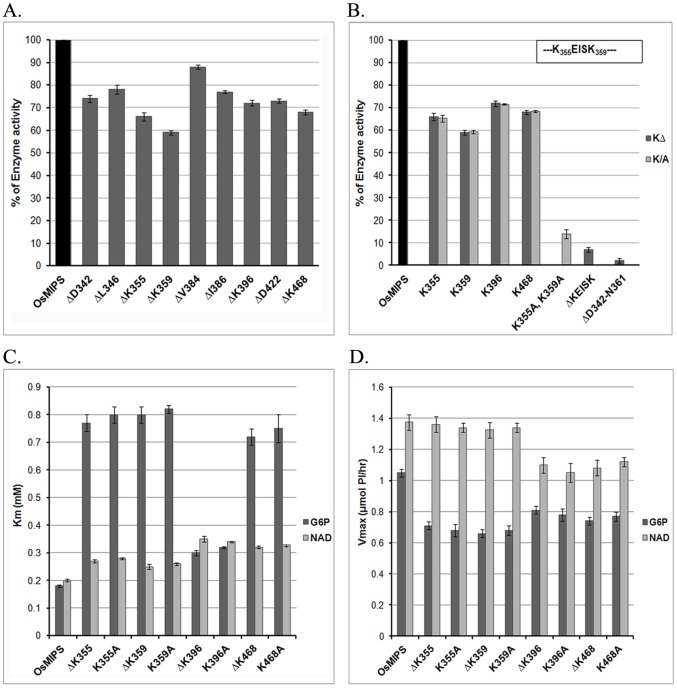
Comparison of enzyme activity of wild *Os*MIPS protein and its mutants. (A) Residual MIPS activity of deletion mutants with respect to the wild type enzyme *Os*MIPS. Black arrows indicate the four lysine deletion mutants having least enzyme activity. (B) Residual MIPS activity of deletion and replacement mutants of lysine residues within the catalytic domain represented as KΔ and K/A, respectively. ΔKEISK represents the pentapeptide (inset) deletion mutant while K355A, K359A represents double substitution at positions 355 and 359 of *Os*MIPS. (C) Km and (D) Vmax values for the substrate, G6P and cofactor, NAD, as calculated from Lineweaver-Burk plot.

Although none of the mutants is completely inactive, a variation in degree of reduction in the functional activity amongst them were observed for all nine point deletion mutants with an almost complete loss of activity when the catalytic stretch between D342—N361 was deleted (Fig 5A and 5B). The reduction of enzymatic activity remained unchanged either for the Lys deleted or Lys substituted mutant proteins when compared with the wild *Os*MIPS protein. As for the other mutants, the enzyme activities were variable e.g. ~73% for ΔD422, ~90% and ~79% for ΔV384 and ΔI386 mutants, ~75% and ~78% for ΔD342 and ΔL346 as compared to that of the wild type enzyme (Fig 5A). In comparison, minimum activity for point mutants was observed at positions K355 and K359. Only ~14% activity was noticed in the double mutant K355A, K359A while only an activity of ~7% was obtained in ΔKEISK where both K355 to K359 have been removed (Fig 5B).

Further comparison of Km and Vmax values showed a degree of difference as represented in Fig 5(C) and 5(D). The deletion mutant of K355, the first lysine of Block-C, retained ~70% activity compared to wild-type enzyme. Moreover, the Km for G6P has increased with a reduced Vmax value. This points toward decrease in affinity for the substrate although the kinetics for NAD^+^ remains unaltered. And even when more substrate and cofactor were added, the rate of reaction never reached the Vmax comparable to wild *Os*MIPS. Similarly, mutant of the second lysine of Block-C, ΔK359, retained only ~59% activity. Enzyme kinetics studies showed that the Km for G6P is about four fold higher than the wild type *Os*MIPS. The Vmax has been considerably reduced as well. However, the Km and Vmax for NAD^+^ is quite comparable to that of wild *Os*MIPS. The ΔK396 mutant of the invariant lysine of Block-B was found to retain ~75% activity. A considerable reduction in affinity for the substrate was noticed as from the data obtained from Km and Vmax for the substrate, G6P. The Km for the substrate has increased approximately two fold with decreased Vmax value. The ΔK468 mutant of a lysine outside the predicted conserved domains, maintained ~72% activity compared to wild-type *Os*MIPS. The Km for the substrate showed nearly fourfold increase. Both the ΔK396 and ΔK468 mutants showed considerably unaltered Km and Vmax for the cofactor. Almost identical results in terms of Km and Vmax were obtained for the substitution mutants for the indicated Lys residues (Fig 5C and 5D).

Contrary to the report by Jin *et al*. [15], no completely defunct mutant for *Os*MIPS was observed by deletion of any of the critical lysine residues. It is possible that upon encapsulation of the substrate molecule, the relative physical distance between the lysine residues were shortened. As a result, upon deletion of a particular lysine, the functional activity has been affected but not to an extent of complete abolition of activity and the other residues still can maintain a residual activity. However, complete loss of activity in the mutant ΔOsINO1-D342-N361 [40] and a minimal activity as observed for pentapeptide (KEISK) deletion and the double mutant enzyme, the present study suggests that the two lysine residues belonging to the conserved block-C, f-r-S-K-e-i-s-K-x are certainly essential to the functional activity of *Os*MIPS.

### The presence of the conserved pentapeptide K-x-x-x-K at the active pocket of MIPS indicate strong structure-to-function correlation

**Fig 6:**
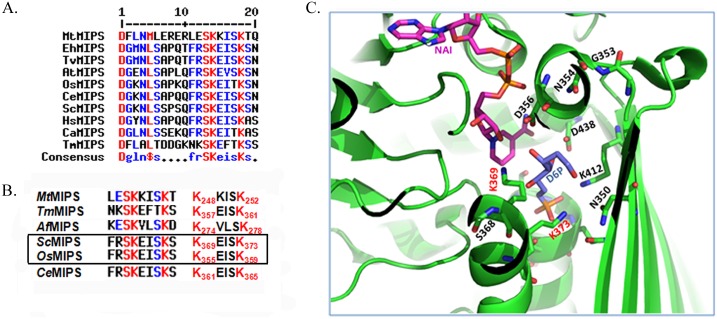
Phylogenetic conservation of the pentapeptide, K-x-x-x-K. (A) MultAlin of the 'core catalytic domain’ among representative MIPS sequences from each phyla- *Mycobacterium tuberculosis*, *Thermotoga maritima*, *Saccharomyces cerevisiae*, *Candida albicans*, *Entamoeba histolytica*, *Trypanosoma vivax*, *Oryza sativa*, *Arabidopsis thaliana*, *Caenorhabditis elegans* and *Homo sapiens* showing 100% conservation of the two lysine residues belonging to pentapeptide. (B) Loci of the conserved lysine residues in the MIPS sequences in the established crystal structures, *Mt*MIPS, *Tm*MIPS *Af*MIPS, *Sc*MIPS and *Ce*MIPS compared with MIPS from *Oryza sativa*, (*Os*MIPS). As per SWISSMODEL, *Os*MIPS protein structure closely resembles *Sc*MIPS, shown within black bounding box. (C) The conserved amino acids with ≥90% similarity within the four conserved blocks of catalytic domain are marked on yeast MIPS crystal structure, 1RM0 complexed with inhibitor, D6P and cofactor, NADH. The two lysine residues belonging to conserved pentapeptide are represented in red font. The figure has been generated using PyMOL. To make the residues of the catalytic core visible, some of the residues have been removed from the figure.

The block-C at the active site of MIPS was found to invariably conserve FRSKEISKS in all the plant sequences. A protein modelling was performed for *Os*MIPS using SWISS-MODEL and since there is no model yet reported from plants, the model was formed using MIPS crystal structures from yeast, 1rm0.1, as the template with 54.65% of sequence identity and GMQE (Global Model Quality Estimation) value of 0.80. Thus, all the conserved amino acids belonging to catalytic domains- N350, G353, N354, D356, S368, K369, K373, K412 and D438 were labelled onto a yeast crystal model 1RM0 complexed with NAD^+^ and an high-affinity inhibitor, 2-deoxy-D-glucitol 6-(E)-vinylhomophosphonate (D6P), reported by Jin *et al*. [15] (Fig 6). As is predicted from the Fig 6C, among all the 9 residues studied, K369, K373, K412 and D438 were found to directly interact with the substrate whereas, D356 and K369 interact with NADH.

The lysine residues, K369 and K373, belonging to the conserved pentapeptide stretch in *Sc*MIPS (Fig 6B), has been reported to function in charge stabilisation at O5 and O1 respectively. K373 is also involved in protonation [15]. Therefore, the two corresponding lysine residues from the conserved motif K-x-x-x-K of *Os*MIPS, K355 and K359, are also likely to participate similarly in substrate binding or catalysis. It is thus apparent that despite the active site similarity between the yeast and the rice enzyme (as judged by the superimposition of rice sequences onto the yeast model), behaviour of the mutants with respect to the activities and kinetic properties seem to be different. This intriguing observation can be resolved only with the actual structure determination of the rice enzyme.

## Conclusion

Conversion of the amino acid residues constituting a protein based on biochemical properties of the side chain helped in identifying six evolutionary conserved blocks among divergent eukaryotic MIPS. However, there are few conservative amino acids present within conservative mutations in each of the blocks identified which have restricted evolutionary rate to maintain the characteristic functionality of the protein. One of the evolutionarily conserved blocks, a 9-mer peptide stretch, f-r-S-K-e-i-s-K-x, has been identified at the catalytic site of MIPS throughout evolution from unicellular bacteria to a complex multicellular organism. This conserved nanopeptide has two invariant lysine residues separated by a three amino acid spacer forming the highly conserved pentapeptide, K-x-x-x-K. Functional validation of the significance of the two lysine residues within this block have been achieved through *in vitro* mutagenesis of a MIPS from a monocot model plant, *Oryza sativa*, followed by the analysis of the change in functional activity of the mutants.

## Supporting information

S1 Table MIPS homologous sequences selected for evolutionary analysis. The homologous sequences have been arranged kingdom-wise.
(DOCX)

S1 Fig Effect of Lys modification on *Os*MIPS. (A)Free amino groups (Lys) in *Os*MIPS was calculated by adding 110μl of 0.1% aqueous solution of TNBS to 300μl purified protein solution 30minutes in dark at 25°C as described in Material and method section. The reaction was terminated with 2.5% SDS and 0.1(N) HCl. A proper blank (no enzyme set) as well as a negative control (–TNBS) were also treated similarly. Spectra of the samples were taken from 300nm to 400nm and absorbance plotted against the wavelength. The absorbance spectrum showed peak at 367nm from which free amino group in the protein was calculated. (B) A plot of % residual synthase activity of MIPS treated with TNBS (□) as described in Materials and Methods section compared to TNBS untreated MIPS (●) under the same experimental conditions.
(TIF)
