# Supplementary material for: An evolutionary analysis identifies a conserved pentapeptide stretch containing the two essential lysine residues for rice L-myo-inositol 1-phosphate synthase catalytic activity
Source: PLoS One. 2017 Sep 26;12(9):e0185351. doi: 10.1371/journal.pone.0185351 (PMC5614600; doi:10.1371/journal.pone.0185351)
Supplement: S1 Table — The homologous sequences have been arranged kingdom-wise. (DOCX) [file pone.0185351.s001.docx]

**S1 Table. MIPS homologous sequences selected for evolutionary analysis.** The homologous sequences have been arranged kingdom-wise.

| **Kingdom** | **Seq. No** | **Codes used** | **Name of the organism** | **Accession Number** | **Seq. Length** |
| --- | --- | --- | --- | --- | --- |
| **Bacteria** | 1 | Ba1 | *Clostridium sp.* D5 | WP_009002129.1 | 358 |
|  | 2 | Ba2 | *Streptomyces griseus* subsp. *griseus* | KUL57401.1 | 360 |
|  | 3 | Ba3 | *Bacillus thuringiensis* | WP_000794578.1 | 363 |
|  | 4 | Ba4 | *Mycobacterium tuberculosis* | WP_003902822.1 | 367 |
|  | 5 | Ba5 | *Rhizobium leguminosarum* | WP_025395215.1 | 367 |
|  | 6 | Ba6 | *Thermotoga maritima* | AKE27308.1 | 382 |
| **Archaea** | 7 | Ar1 | *Metallosphaera cuprina* Ar-4 | AEB94641.1 | 358 |
|  | 8 | Ar2 | *Sulfolobus acidocaldarius* DSM 639 | AAY80749.1 | 358 |
|  | 9 | Ar3 | *Metallosphaera sedula* DSM 5348 | ABP95848.1 | 359 |
|  | 10 | Ar4 | *Sulfolobus islandicus* HVE10/4 | ADX82628.1 | 359 |
|  | 11 | Ar5 | *Sulfolobus islandicus* L.D.8.5 | ADB87114.1 | 359 |
|  | 12 | Ar6 | *Sulfolobus islandicus* L.S.2.15 | ACP35429.1 | 359 |
|  | 13 | Ar7 | *Sulfolobus islandicus* LAL14/1 | AGJ62642.1 | 359 |
|  | 14 | Ar8 | *Sulfolobus islandicus* M.14.25 | ACP38088.1 | 359 |
|  | 15 | Ar9 | *Sulfolobus islandicus* M.16.27 | ACP55267.1 | 359 |
|  | 16 | Ar10 | *Sulfolobus islandicus* M.16.4 | ACR41922.1 | 359 |
|  | 17 | Ar11 | *Sulfolobus islandicus* REY15A | ADX85264.1 | 359 |
|  | 18 | Ar12 | *Sulfolobus islandicus* Y.G.57.14 | ACP45595.1 | 359 |
|  | 19 | Ar13 | *Sulfolobus islandicus* Y.N.15.51 | ACP48617.1 | 359 |
|  | 20 | Ar14 | *Sulfolobus solfataricus* 98/2 | ACX92080.1 | 359 |
|  | 21 | Ar15 | *Sulfolobus tokodaii* str. 7 | BAB66276.1 | 359 |
|  | 22 | Ar16 | *Acidianus hospitalis* W1 | AEE94239.1 | 361 |
|  | 23 | Ar17 | *Candidatus korarchaeum cryptofilum* OPF8 | ACB07812.1 | 361 |
|  | 24 | Ar18 | *Candidatus nitrosoarchaeum limnia* SFB1 | EGG42902.1 | 364 |
|  | 25 | Ar19 | *Methanobrevibacter ruminantium* M1 | ADC47737.1 | 364 |
|  | 26 | Ar20 | *Methanobrevibacter smithii* ATCC 35061 | ABQ87145.1 | 364 |
|  | 27 | Ar21 | *Methanobrevibacter smithii* DSM 2374 | EFC93288.1 | 364 |
|  | 28 | Ar22 | *Methanobrevibacter smithii* DSM 2375 | EEE41967.1 | 364 |
|  | 29 | Ar23 | *Nitrosopumilus maritimus* SCM1 | ABX12545.1 | 364 |
|  | 30 | Ar24 | *Thaumarchaeota archaeon* CSP1-1 | KRT61425.1 | 364 |
|  | 31 | Ar25 | *Methanothermus fervidus* DSM 2088 | ADP77437.1 | 365 |
|  | 32 | Ar26 | *Methanosarcina barkeri* str. Fusaro | AAZ70008.1 | 366 |
|  | 33 | Ar27 | *Methanosarcina mazei* Go1 | AAM31066.1 | 366 |
|  | 34 | Ar28 | *Methanococcoides burtonii* DSM 6242 | ABE53233.1 | 367 |
|  | 35 | Ar29 | *Methanoculleus marisnigri* JR1 | ABN56907.1 | 367 |
|  | 36 | Ar30 | *Methanohalobium evestigatum* Z-7303 | ADI74978.1 | 367 |
|  | 37 | Ar31 | *Methanohalophilus mahii* DSM 5219 | ADE37020.1 | 367 |
|  | 38 | Ar32 | *Methanosalsum zhilinae* DSM 4017 | AEH61601.1 | 367 |
|  | 39 | Ar33 | *Methanosaeta thermophila* PT | ABK14073.1 | 369 |
|  | 40 | Ar34 | *Ignisphaera aggregans* DSM17230 | ADM28082.1 | 375 |
|  | 41 | Ar35 | *Staphylothermus marinus* F1 | ABN69411.1 | 377 |
|  | 42 | Ar36 | *Methanosaeta concilii* GP6 | AEB66950.1 | 379 |
|  | 43 | Ar37 | *Desulfurococcus kamchatkensis* 1221n | ACL11235.1 | 380 |
|  | 44 | Ar38 | *Haloferax volcanii* | WP_049914735.1 | 380 |
| **Fungi** | 45 | Fu1 | *Saccharomyces cerevisiae* S288c | NP_012382.2 | 533 |
|  | 46 | Fu2 | *Verticillium dahliae* VdLs.17 | EGY13359.1 | 503 |
|  | 47 | Fu3 | *Coccidioides immitis* RMSCC 3703 | KMU75761.1 | 507 |
|  | 48 | Fu4 | *Candida tropicalis* MYA-3404 | EER30429.1 | 517 |
|  | 49 | Fu5 | *Lodderomyces elongisporus* NRRL YB-4239 | EDK47101.1 | 518 |
|  | 50 | Fu6 | *Tolypocladium ophioglossoides* CBS 100239 | KND89758.1 | 519 |
|  | 51 | Fu7 | *Debaryomyces fabryi* | KSA04114.1 | 520 |
|  | 52 | Fu8 | *Cyberlindnera jadinii* | CEP25255.1 | 520 |
|  | 53 | Fu9 | *Wickerhamomyces ciferrii* | CCH44319.1 | 520 |
|  | 54 | Fu10 | *Talaromyces stipitatus* ATCC 10500 | EED20870.1 | 520 |
|  | 55 | Fu11 | *Candida albicans* SC5314 | KHC69745.1 | 520 |
|  | 56 | Fu12 | *Candida dubliniensis* CD36 | CAX40478.1 | 520 |
|  | 57 | Fu13 | *Candida orthopsilosis* | CCG22921.1 | 520 |
|  | 58 | Fu14 | *Candida orthopsilosis* Co90*-125* | XP_003869058.1 | 520 |
|  | 59 | Fu15 | *Candida tenuis* ATCC 10573 | EGV66846.1 | 521 |
|  | 60 | Fu16 | *Cyberlindnera fabianii* | CDR38016.1 | 522 |
|  | 61 | Fu17 | *Ogataea parapolymorpha* DL-1 | ESX01855.1 | 523 |
|  | 62 | Fu18 | *Komagataella pastoris* | AAC33791.1 | 525 |
|  | 63 | Fu19 | *Komagataella phaffii* CBS7435 | CCA37816.1 | 525 |
|  | 64 | Fu20 | *Komagataella phaffii* GS115 | CAY69813.1 | 525 |
|  | 65 | Fu21 | *Mitosporidium daphniae* | KGG52942.1 | 526 |
|  | 66 | Fu22 | *Mortierella verticillata* NRRL6337 | KFH65357.1 | 526 |
|  | 67 | Fu23 | *Mortierella verticillata* NRRL6337 | KFH71508.1 | 526 |
|  | 68 | Fu24 | *Rhizophagus irregularis* DAOM 197198w | EXX75657.1 | 526 |
|  | 69 | Fu25 | *Conidiobolus coronatus* NRRL 28638 | KXN67460.1 | 527 |
|  | 70 | Fu26 | *Wallemia ichthyophaga* EXF-994 | EOR04044.1 | 529 |
|  | 71 | Fu27 | *Wallemia mellicola* CBS 633.66 | EIM23705.1 | 529 |
|  | 72 | Fu28 | *Ceratocystis platani* | KKF97177.1 | 529 |
| **Protista** | 73 | Pr1 | *Entamoeba dispar* SAW760 | XP_001737288.1 | 508 |
|  | 74 | Pr2 | *Entamoeba histolytica* HM-1:IMSS | XP_654310.1 | 508 |
|  | 75 | Pr3 | *Polysphondylium pallidum* PN500 | EFA77141.1 | 511 |
|  | 76 | Pr4 | *Dictyostelium discoideum* AX4 | XP_638094.1 | 511 |
|  | 77 | Pr5 | *Dictyostelium purpureum* | XP_003291265.1 | 511 |
|  | 78 | Pr6 | *Bodo saltans* | CUG92382.1 | 512 |
|  | 79 | Pr7 | *Plasmopara halstedii* | CEG42668.1 | 517 |
|  | 80 | Pr8 | *Saprolegnia diclina* VS20 | XP_008605672.1 | 517 |
|  | 81 | Pr9 | *Saprolegnia parasitica* CBS 223.65 | XP_012201001.1 | 517 |
|  | 82 | Pr10 | *Thecamonas trahens* ATCC 50062 | XP_013762089.1 | 519 |
|  | 83 | Pr11 | *Gregarina niphandrodes* | XP_011132074.1 | 521 |
|  | 84 | Pr12 | *Acanthamoeba castellanii* strain Neff | XP_004339228.1 | 521 |
|  | 85 | Pr13 | *Angomonas deanei* | AJP61944.1 | 521 |
|  | 86 | Pr14 | *Angomonas desouzai* | AJP61945.1 | 522 |
|  | 87 | Pr15 | *Naegleria gruberi* strain NEG-M | XP_002678850.1 | 523 |
|  | 88 | Pr16 | *Leishmania braziliensis* MHOM/BR/75/M2904 | XP_001563439.1 | 526 |
|  | 89 | Pr17 | *Leishmania major* strain Friedlin | XP_001687736.1 | 526 |
|  | 90 | Pr18 | *Leishmania mexicana* MHOM/GT/2001/U1103 | XP_003873511.1 | 526 |
|  | 91 | Pr19 | *Leishmania panamensis* | XP_010697365.1 | 526 |
|  | 92 | Pr20 | *Leptomonas seymouri* | KPI83934.1 | 526 |
|  | 93 | Pr21 | *Leishmania amazonensis* | AAB51376.1 | 527 |
|  | 94 | Pr22 | *Trypanosoma vivax*Y486 | CCC51699.1 | 527 |
|  | 95 | Pr23 | *Trypanosoma brucei brucei* TREU927 | XP_822946.1 | 528 |
|  | 96 | Pr24 | *Trypanosoma cruzi* strain CL Brener | XP_813077.1 | 529 |
| **Planta** | 97 | Pl1 | *Chlamydomonas reinhardtii* | EDP05930.1 | 507 |
|  | 98 | Pl2 | *Oryza sativa* subsp. *japonica* (Os10g0369900) | BAT10553.1 | 509 |
|  | 99 | Pl3 | *Helicosporidium sp.* ATCC 50920 | KDD74931.1 | 509 |
|  | 100 | Pl4 | *Zea mays* | NP_001105552.1 | 509 |
|  | 101 | Pl5 | *Avicennia marina* | AAK21969.1 | 509 |
|  | 102 | Pl6 | *Coffea arabica* | ACZ57952.1 | 510 |
|  | 103 | Pl7 | *Medicago truncatula* | XP_003601987.1 | 510 |
|  | 104 | Pl8 | *Triticum aestivum* | AAD26330.1/ AF120146_1 | 510 |
|  | 105 | Pl9 | *Camellia sinensis* | AJO70149.1 | 510 |
|  | 106 | Pl10 | *Ricinus communis* | NP_001310667.1 | 510 |
|  | 107 | Pl11 | *Cicer arietinum* | NP_001266035.1 | 510 |
|  | 108 | Pl12 | *Nicotiana tabacum* | BAA95788.1 | 510 |
|  | 109 | Pl13 | *Cynara cardunculus* var*. scolymus* | KVH41929.1 | 510 |
|  | 110 | Pl14 | *Hordeum vulgare* subsp. *vulgare* | AAC17133.1 | 510 |
|  | 111 | Pl15 | *Dorcoceras hygrometricum* | KZV56787.1 | 510 |
|  | 112 | Pl16 | *Setaria italica* | NP_001267776.1 | 510 |
|  | 113 | Pl17 | *Hevea brasiliensis* | AFD61599.1 | 510 |
|  | 114 | Pl18 | *Oryza sativa* subsp. *japonica* (Os03g0192700) | BAS82735.1 | 510 |
|  | 115 | Pl19 | *Klebsormidium flaccidum* | GAQ90006.1 | 510 |
|  | 116 | Pl20 | *Nicotiana paniculata* | BAA84084.1 | 510 |
|  | 117 | Pl21 | *Avena sativa* | BAB40956.2 | 510 |
|  | 118 | Pl22 | *Passiflora edulis f. flavicarpa* | ABF51620.1 | 510 |
|  | 119 | Pl23 | *Phaseolus vulgaris* | CAX94843.1 | 510 |
|  | 120 | Pl24 | *Brassica napus* | NP_001302750.1 | 510 |
|  | 121 | Pl25 | *Saccharum officinarum* | ALO50702.1 | 510 |
|  | 122 | Pl26 | *Saccharum spontaneum* | ALO50704.1 | 510 |
|  | 123 | Pl27 | *Sesamum indicum* | NP_001291338.1 | 510 |
|  | 124 | Pl28 | *Medicago falcata* | ABO77439.1 | 510 |
|  | 125 | Pl29 | *Glycine max* | NP_001235249.1 | 510 |
|  | 126 | Pl30 | *Suaeda salsa* | AAL28131.1\|AF433879_1 | 510 |
|  | 127 | Pl31 | *Arabidopsis thaliana* | AAK96645.1 | 510 |
|  | 128 | Pl32 | *Arabidopsis thaliana* | NP_196579.1 | 510 |
|  | 129 | Pl33 | *Gossypium hirsutum* | ACJ11714.1 | 510 |
|  | 130 | Pl34 | *Citrullus lanatus* subsp*. vulgaris* | BAI52951.1 | 510 |
|  | 131 | Pl35 | *Xerophyta viscosa* | AAP85531.1 | 510 |
|  | 132 | Pl36 | *Arabidopsis thaliana* | AAK50093.1 | 511 |
|  | 133 | Pl37 | *Trifolium repens* | ADD09590.1 | 511 |
|  | 134 | Pl38 | *Mesembryanthemum crystallinum* | AAB03687.1 | 512 |
|  | 135 | Pl39 | *Oryza coarctata* | AAP74579.1 | 512 |
|  | 136 | Pl40 | *Bathycoccus prasinos* | XP_007509183.1 | 520 |
| **Animalia** | 137 | An1 | *Caenorhabditis elegans* | NP_496499.2 | 525 |
|  | 138 | An2 | *Tribolium castaneum* | EFA09280.1 | 526 |
|  | 139 | An3 | *Thelohanellus kitauei* | KII63896.1 | 529 |
|  | 140 | An4 | *Ophiophagus hannah* | ETE66525.1 | 537 |
|  | 141 | An5 | *Rhipicephalus appendiculatus* | JAP86502.1 | 538 |
|  | 142 | An6 | *Papilio xuthus* | KPJ02325.1 | 539 |
|  | 143 | An7 | *Crassostrea gigas* | EKC35396.1 | 539 |
|  | 144 | An8 | *Fundulus heteroclitus* | JAR07412.1 | 539 |
|  | 145 | An9 | *Callorhinchus milii* | NP_001279807.1 | 545 |
|  | 146 | An10 | *Strongyloides ratti* | CEF60401.1 | 545 |
|  | 147 | An11 | *Papilio machaon* | KPJ12797.1 | 548 |
|  | 148 | An12 | *Ancylostoma ceylanicum* | EPB76710.1 | 550 |
|  | 149 | An13 | *Salmo salar* | NP_001133802.1 | 551 |
|  | 150 | An14 | *Culex quinquefasciatus* | XP_001848345.1 | 551 |
|  | 151 | An15 | *Myotis brandtii* | EPQ12449.1 | 552 |
|  | 152 | An16 | *Cyprinus carpio* | AER39749.1 | 552 |
|  | 153 | An17 | *Tupaia chinensis* | ELW67149.1 | 553 |
|  | 154 | An18 | *Anopheles darlingi* | ETN64673.1 | 554 |
|  | 155 | An19 | *Fundulus heteroclitus* | JAR82477.1 | 555 |
|  | 156 | An20 | *Pteropus alecto* | ELK19191.1 | 556 |
|  | 157 | An21 | *Bos taurus* | NP_001039497.1 | 557 |
|  | 158 | An22 | *Mus musculus* | NP_076116.1 | 557 |
|  | 159 | An23 | *Rattus norvegicus* | NP_001013902.2 | 557 |
|  | 160 | An24 | *Pan troglodytes* | BAK61977.1 | 558 |
|  | 161 | An25 | *Cricetulus griseus* | ERE91131.1 | 558 |
|  | 162 | An26 | *Homo sapiens* | AAF26444.1 | 558 |
|  | 163 | An27 | *Macaca fascicularis* | NP_001270531.1 | 559 |
|  | 164 | An28 | *Musca domestica* | AFP61799.1 | 559 |
|  | 165 | An29 | *Larimichthys crocea* | KKF13767.1 | 559 |
|  | 166 | An30 | *Macaca mulatta* | NP_001248248.1 | 559 |
|  | 167 | An31 | *Lucilia cuprina* | KNC24206.1 | 561 |
|  | 168 | An32 | *Xenopus laevis* | NP_001079540.1 | 563 |
|  | 169 | An33 | *Xenopus laevis* | NP_001086783.2 | 563 |
|  | 170 | An34 | *Zootermopsis nevadensis* | KDR18213.1 | 563 |
|  | 171 | An35 | *Xenopus tropicalis* | NP_001119985.1 | 564 |
|  | 172 | An36 | *Drosophila melanogaster* | AAD02819.1 | 565 |
