# Supplementary figures and images for: An evolutionary analysis identifies a conserved pentapeptide stretch containing the two essential lysine residues for rice L-myo-inositol 1-phosphate synthase catalytic activity
Source: PLoS One. 2017 Sep 26;12(9):e0185351. doi: 10.1371/journal.pone.0185351 (PMC5614600; doi:10.1371/journal.pone.0185351)

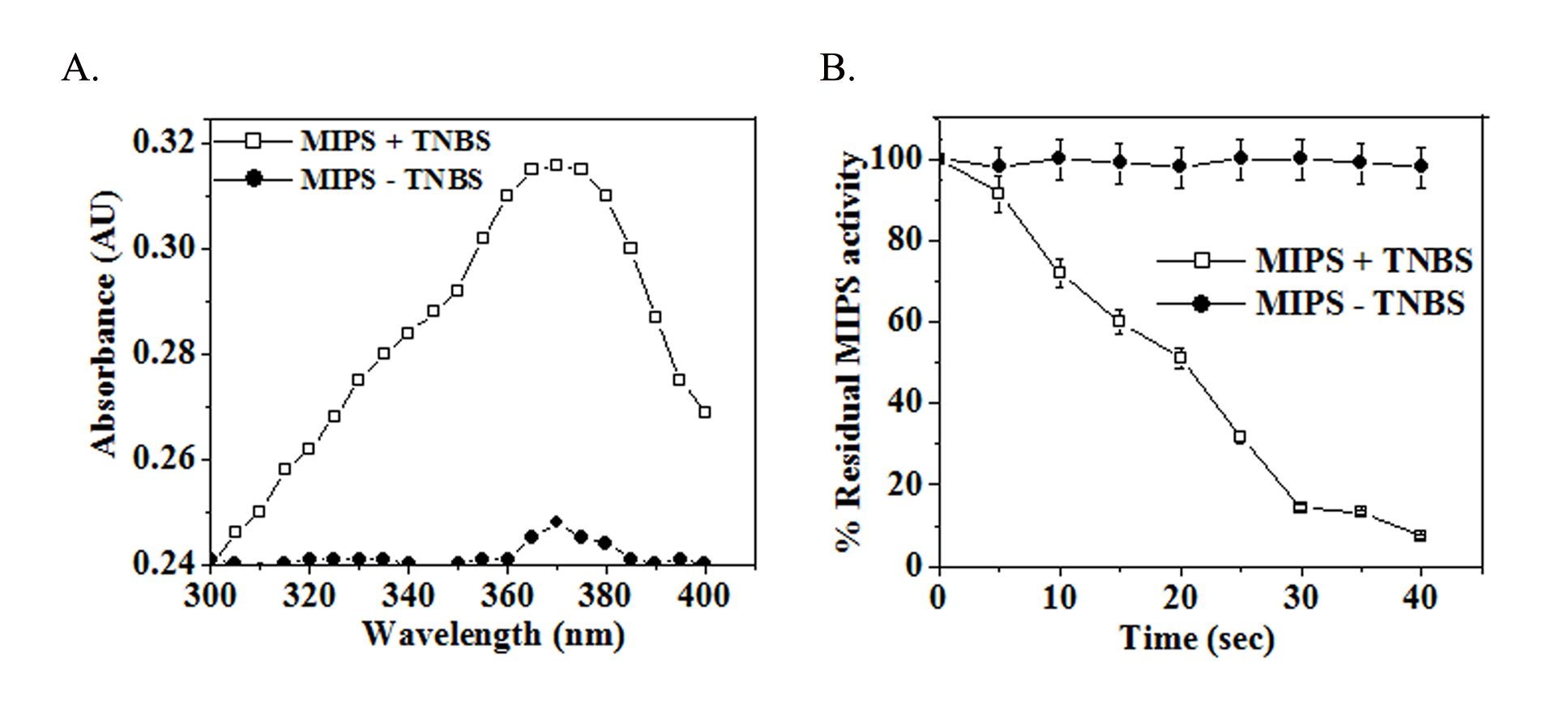

Supplement: S1 Fig — (A)Free amino groups (Lys) in OsMIPS was calculated by adding 110μl of 0.1% aqueous solution of TNBS to 300μl purified protein solution 30minutes in dark at 25°C as described in Material and method section. The reaction was terminated with 2.5% SDS and 0.1(N) HCl. A proper blank (no enzyme set) as well as a negative control (–TNBS) were also treated similarly. Spectra of the samples were taken from 300nm to 400nm and absorbance plotted against the wavelength. The absorbance spectrum showed peak at 367nm from which free amino group in the protein was calculated. (B) A plot of % residual synthase activity of MIPS treated with TNBS (□) as described in Materials and Methods section compared to TNBS untreated MIPS (●) under the same experimental conditions. (TIF) [file pone.0185351.s002.tif]
